# Sequence Variation of Epstein-Barr Virus: Viral Types, Geography, Codon Usage, and Diseases

**DOI:** 10.1128/JVI.01132-18

**Published:** 2018-10-29

**Authors:** Samantha Correia, Ray Bridges, Fanny Wegner, Cristina Venturini, Anne Palser, Jaap M. Middeldorp, Jeffrey I. Cohen, Mario A. Lorenzetti, Irene Bassano, Robert E. White, Paul Kellam, Judith Breuer, Paul J. Farrell

**Affiliations:** a Section of Virology, Faculty of Medicine, Norfolk Place, London, United Kingdom; b Division of Infection and Immunity, University College London, London, United Kingdom; c Wellcome Trust Sanger Institute, Hinxton, Cambridge, United Kingdom; d Department of Pathology, VU University Medical Center, Amsterdam, Netherlands; e Laboratory of Infectious Diseases, National Institute of Allergy and Infectious Diseases, Bethesda, Maryland, USA; f Instituto Multidisciplinario de Investigaciones en Patologías Pediátricas (IMIPP), CONICET-GCBA, División Patología, Hospital de Niños Ricardo Gutiérrez, Buenos Aires, Argentina; Northwestern University

**Keywords:** Epstein-Barr virus

## Abstract

One hundred thirty-eight new Epstein-Barr virus (EBV) genome sequences have been determined. One hundred twenty-five of these and 116 from previous reports were combined to produce a multiple-sequence alignment of 241 EBV genomes, which we have used to analyze variation within the viral genome. The type 1/type 2 classification of EBV remains the major form of variation and is defined mostly by EBNA2 and EBNA3, but the type 2 single-nucleotide polymorphisms (SNPs) at the EBNA3 locus extend into the adjacent gp350 and gp42 genes, whose products mediate infection of B cells by EBV. A small insertion within the BART microRNA region of the genome was present in 21 EBV strains. EBV from saliva of U.S. patients with chronic active EBV infection aligned with the wild-type EBV genome with no evidence of WZhet rearrangements. The V3 polymorphism in the Zp promoter for BZLF1 was found to be frequent in nasopharyngeal carcinoma cases from both Hong Kong and Indonesia. Codon usage was found to differ between latent and lytic cycle EBV genes, and the main forms of variation of the EBNA1 protein have been identified.

**IMPORTANCE** Epstein-Barr virus causes most cases of infectious mononucleosis and posttransplant lymphoproliferative disease. It contributes to several types of cancer, including Hodgkin's lymphoma, Burkitt's lymphoma, diffuse large B cell lymphoma, nasopharyngeal carcinoma, and gastric carcinoma. EBV genome variation is important because some of the diseases associated with EBV have very different incidences in different populations and geographic regions, and differences in the EBV genome might contribute to these diseases. Some specific EBV genome alterations that appear to be significant in EBV-associated cancers are already known, and current efforts to make an EBV vaccine and antiviral drugs should also take account of sequence differences in the proteins used as targets.

## Abstract (precis)

Epstein-Barr virus causes most cases of infectious mononucleosis and posttransplant lymphoproliferative disease. It contributes to several types of cancer, including Hodgkin's lymphoma, Burkitt's lymphoma, diffuse large B cell lymphoma, nasopharyngeal carcinoma, and gastric carcinoma. EBV genome variation is important because some of the diseases associated with EBV have very different incidences in different populations and geographic regions, and differences in the EBV genome might contribute to these diseases. Some specific EBV genome alterations that appear to be significant in EBV-associated cancers are already known, and current efforts to make an EBV vaccine and antiviral drugs should also take account of sequence differences in the proteins used as targets.

## INTRODUCTION

Most of the world's population is thought to be infected by Epstein-Barr virus (EBV) without any symptoms or disease. However, some people suffer from infectious mononucleosis after primary infection, and EBV is also present in the malignant cells of several types of cancer, where it appears to contribute to the pathogenesis of the disease (1). Some of the EBV-associated diseases differ significantly in incidence in different parts of the world, raising the possibility that variation in the genome of EBV is relevant to some of these diseases.

There is already some evidence for mutations in EBV being linked to certain types of cancer. For example, about 10% of African Burkitt's lymphoma (BL) cases contain EBV with a deletion of the EBNA2 locus, which has been proposed to result in a higher expression of the EBV BHRF1 homologue of the antiapoptosis BCL2 protein (2). Also, mutations in the EBNA3B gene have been shown to make EBV more oncogenic, causing disease similar to diffuse large B cell lymphoma (DLBCL) in a mouse model system, and examples of mutated EBNA3B have been found in human EBV-associated DLBCL cases (3). However, identifying EBV mutations in cancers requires a detailed understanding of the background variation in the normal viral genome so that potentially significant mutations can be distinguished from natural variation in the virus genome.

Variation in the viral genome is also relevant to development of potential vaccines for EBV, where strain differences have already been identified in the relevant part of the gp350 protein (4), which is a key component of current vaccines.

In recent reports we analyzed variation in some key genes of EBV using a combination of templated and *de novo*-assembled viral genomes (4, 5). Here, we present the new *de novo*-assembled genome sequences of 138 EBV strains and use these genomes and previously published sequences to identify major patterns of variation. We explore single-nucleotide polymorphisms (SNPs) which have been proposed to be related to EBV-associated diseases, infections with multiple EBV strains, examples of genetic linkage within the EBV genome, and differences in codon usage between latent and lytic cycle genes.

## RESULTS

### One hundred thirty-eight new EBV genome sequences and a multiple-sequence alignment of 241 EBV genomes.

Here, we report new *de novo* assemblies of 138 EBV genome sequences. A total of 125 of these and 116 previously published *de novo*-assembled EBV genomes were used to create a multiple-sequence alignment (MSA) of 241 EBV genomes. We used this to analyze major patterns of variation in the virus genome. Table S1 in the supplemental material lists all of the EBV genome sequences used in this paper, with accession numbers and details of the samples from which they were derived. In addition to the 241 aligned EBV genomes, Table S1 includes a small number of additional EBV genome sequences that were omitted from the MSA either because they were essentially duplicates or because they contain rearrangements which would gap the MSA excessively.

The 241 sequences that are analyzed below include 130 that were determined directly from primary material (38 from saliva and 92 from other primary biopsy specimens). Samples came from many different geographic regions of the world; this information is listed in Table S1, and the geographic origin of the sample is represented by colors in the figures where relevant.

### Major patterns of variation are type1/type 2 and geographic.

**FIG 1:**
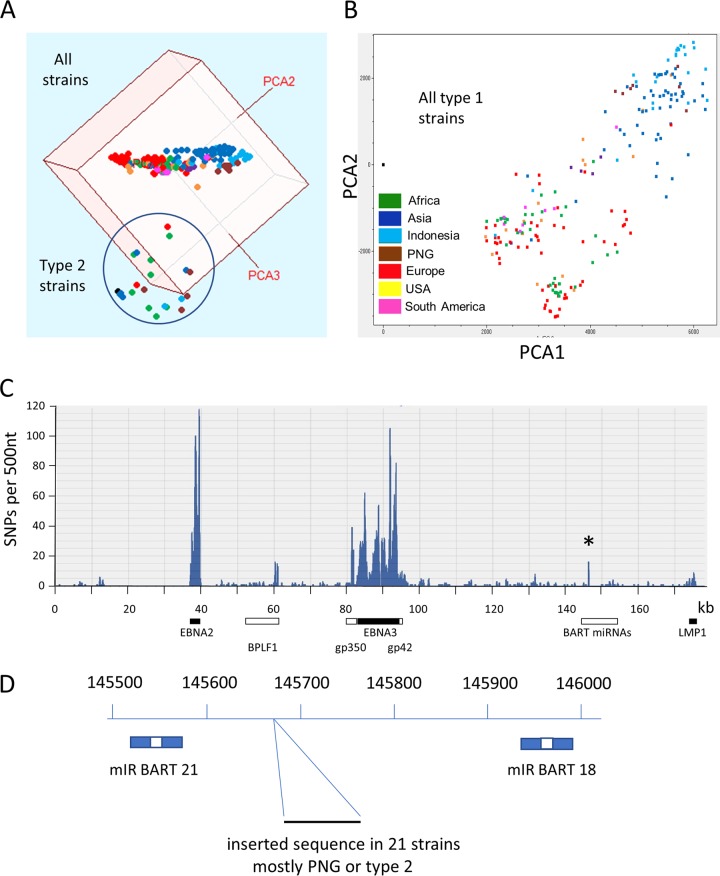
(A) PCA of 241 EBV genomes from the multiple-sequence alignment shown in a three-dimensional plot of PCA1, PCA2, and PCA3. Each genome is represented by a colored dot (geographic color codes are shown in panel B). The cluster of type 2 strains is circled. (B) Plot of PCA1 against PCA2 for the 217 type 1 strains, with each genome sequence represented by a colored dot. The Asian and Indonesian strains cluster away from African and European strains. (C) SNPs of a type 2 consensus genome relative to the type 1 consensus are plotted as number of SNPs in a 500-nt window along the EBV genome (numbered as in the MSA). The positions of EBNA2, BPLF1, gp350, EBNA3, gp42, and LMP1 coding sequences are marked. The peak marked by the asterisk is in the same location as the insertion shown in Fig. 1D. (D) Position of the insertion of 71 to 73 nt in 21 strains in the MSA of 241 EBV genome sequences on a scale numbered as in the NC_007605 reference sequence. The positions of Mir BART 21 and 18 are shown.

Our previous principal component analysis (PCA) of 84 EBV genomes (5) found that type 1/type 2 was the major form of variation and showed that EBNA2 and EBNA3 genes accounted for most of the difference between type 1 and type 2 sequences. Type 1/type 2 remains the largest type of systematic variation in the enlarged set of 241 sequences, which clearly cluster into 2 groups (Fig. 1A) in the first 3 components of the PCA (which account for 45% of the variation in this set of sequences). Comparison of consensus sequences of the type 1 and type 2 genomes with this enlarged data set again showed that SNPs in the regions of EBNA2 and EBNA3 genes are the main differences between type 1 and type 2 (Fig. 1C).

Some features of the PCA procedure used here differ from our previous approach (5). As before, SNPs are determined relative to a consensus of all the sequences, which in practice is close to a consensus of the type 1 sequences because of the smaller number of type 2 genomes analyzed. However, most PCA procedures measure SNPs only in those nucleotide positions which are A, C, T, G, or N in the MSA, ignoring nucleotide positions where there is a deletion in one of the sequences. This tends to reduce the number of positions sampled in MSAs that contain many sequences and might overlook insertions or deletions characteristic of groups of strains. In this new analysis we have modified the PCA to include positions with inserted or deleted nucleotides. To simplify the analysis we have only scored SNPs at positions where more than 5 of the 241 sequences differ from the consensus, taking the view that less frequent SNPs would not affect the results significantly and are unlikely to be biologically significant. Consequently, SNPs at 10,819 positions within the MSA of EBV genomes contributed to the PCA.

Within the type 1 sequences, the first two components of the PCA tended to separate, for example, the Asian (China, Hong Kong, Taiwan, and Japan) (blue), Indonesian (light blue), and Papua New Guinean (brown) strains from the rest (Fig. 1B). For each geographic group there were a few outliers which could now be excluded from the identification of groups of closely related sequences, which we consider here to be characteristic of a geographic region. A phylogenetic tree (Fig. S1) of preliminary templated assemblies (4) of 233 EBV whole-genome sequences (mostly the same strains as those in the MSA of 241 *de novo*-assembled sequences) also helped in this selection process. The EBV sequences which were chosen to represent each geographic group are listed in Table S1. These sequences were used to derive a consensus for each geographic group. Comparison of the consensus sequence for each chosen geographic group and the consensus of all the type 1 sequences did not reveal simple groups of SNPs that were uniquely definitive of each geographic group; instead, a complex mosaic of SNPs determines the geographic differences. Detailed analysis of this will be published elsewhere, but one prominent feature was an insertion of 71 to 73 nucleotides (nt) in a subset of the sequences in the BART region between mIR BART 21 and mIR BART 18 (Fig. 1D). Twenty-one EBV genomes in the MSA contained this insertion (Table S1), and these included several from Papua New Guinea and some type 2 EBV genomes, including AG876, the reference type 2 genome. The small peak marked with an asterisk in Fig. 1C showing SNPs that distinguish EBV type 2 from type 1 corresponds to this location.

### Genetic linkage in the EBV genome.

In contrast to most of the EBV genome, where variation is relatively low, the reference type 1 and type 2 EBNA2 have identical nucleotides at only 51% of the coding sequence, and the reference EBNA3 loci are only identical at 72% of their sequences. The relatively large differences in sequence between the type 1 and type 2 versions of EBNA2 and the EBNA3 genes are considered to prevent homologous recombination between the types at these points and are thus thought to allow the persistence of the alleles in the viral population (6). Although they are separated by over 40 kb of EBV genome on either side in the EBV episome in which extensive historic recombination is generally observed (5), there is strong genetic linkage of the EBNA2 and EBNA3 genes within the types. In the enlarged set of 241 EBV genomes analyzed here, 217 have type 1 EBNA2 and EBNA3, and 22 have type 2 EBNA2 and EBNA3. Only 2 isolates have type 1 EBNA2 with type 2 EBNA3s, and there are no genomes with type 2 and type 1 EBNA2. Therefore, 99% of the EBV genomes show type-specific linkage of EBNA2 and EBNA3 (linkage significant with a *P* value of <0.0001).

**FIG 2:**
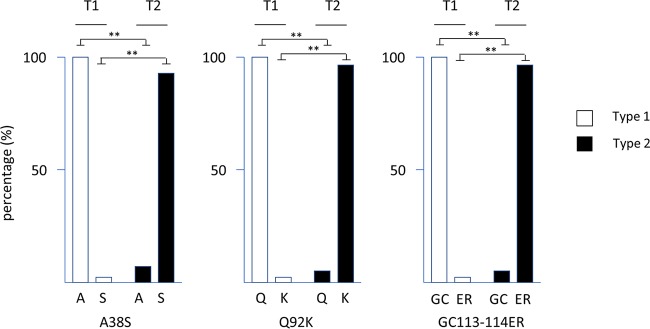
Linkage of gp42 protein sequence variation at the indicated amino acids relative to type 1 or type 2 EBNA3, comparing 24 type 2 EBNA3 protein sequences with 212 type 1 EBNA3 protein sequences. Detailed values and the phylogenetic tree are shown in Fig. S2. Significant differences were determined with an analysis of variance (ANOVA) test. **, *P* < 0.005.

The type-specific sequence at the EBNA3 locus does not stop precisely at the boundaries of the EBNA3 genes, and we reported previously (4) how its effect could be observed in the N terminus of the adjacent gp350 gene (BLLF1), whose protein product mediates EBV infection by binding to CD21 on the B cell. Certain SNPs (including the V3 SNP) in the Zp promoter, which initiates the switch between latency and the viral lytic cycle, were also found to be linked to the type 1/type 2 difference (4). Within this zone of the genome, the gene immediately on the right-hand side of the EBNA3 region on the EBV genome map is the gp42 glycoprotein gene. The gp42 protein also partly mediates B cell infection by EBV through its binding to major histocompatibility complex class II. A phylogenetic tree of gp42 DNA sequences (Fig. S2) clearly segregates the type 2 strains. At the amino acid level, the key differences from type 1 gp42 are A38S, Q92K, G113E, and C114R (Fig. 2). These amino acids are not known to be contact residues of type 1 gp42 with gH or HLA-DR1 (7) but might affect folding or other interactions and be relevant to infection efficiency by type 2 EBV. The sequence differences are also potentially significant for EBV vaccines that may include gp42 as an immunogen (8).

**FIG 3:**
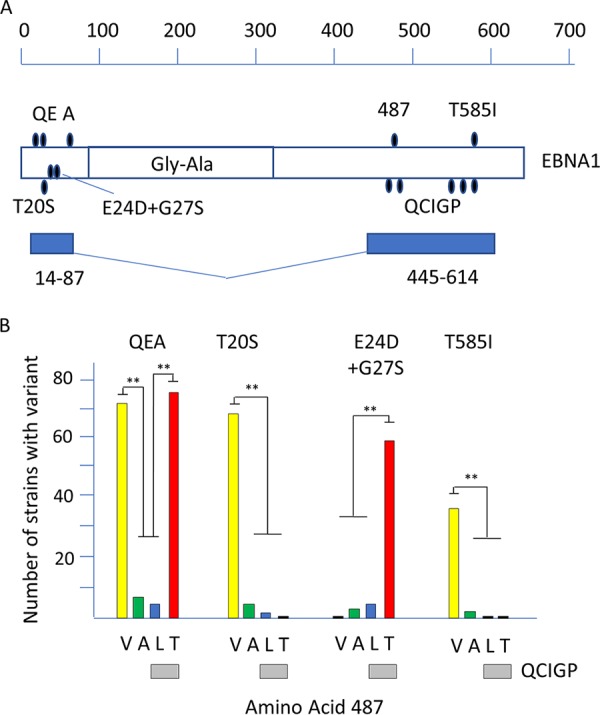
(A) Schematic illustration of locations of the main EBNA1 variant amino acids under a scale of amino acid numbers of B95-8 EBNA1. The Gly-Ala region is marked, and the regions of nt 14 to 87 and 445 to 614, which were concatenated to make the phylogenetic tree (Fig. S3), are shown. (B) Numbers of strains (out of 260) with either the QEA, T20S, E24D+G2S, or T585I variant plotted in relation to amino acid 487 V, A, L, or T. In these EBNA1 sequences, 487 L or T is always found with the QCIGP haplotype. Significant differences were determined with an ANOVA test. **, *P* < 0.005. Full details are shown in Fig. S3.

We previously classified variation in the C-terminal DNA binding domain of EBNA1 into two main groups, determined by variation in 5 amino acids distributed in the sequence; these are PSMVT in the reference strain or QCIGP in many other strains (4). These groups also correlated with variation at amino acid 487, which can be either V, A, L, or T (4). PSMVT strains have V or A at 487, whereas QCIGP strains have L or T at 487 (4). We have now extended that analysis by identifying major patterns of polymorphism in the N-terminal part of EBNA1 and integrating those results with the C-terminal variation (Fig. 3). Even though its protein sequence is divided into two domains by the Gly-Ala repeat sequence (about 229 amino acids in length in B95-8 EBV), there is linkage in variation of the N- and C-terminal domains of EBNA1. To understand the distribution of variation, N-terminal domain amino acids 14 to 87 and C-terminal amino acids 445 to 614 (which contained the relevant polymorphisms) were concatenated, and a resulting phylogenetic tree was labeled with the same color scheme as that used before (4) (Fig. S3). Figure 3A shows the positions of the N-terminal variant amino acids (QEA, T20S, and E24D+G27S) in relation to the previously described C-terminal 487 polymorphisms and the 585 I variant. In Fig. 3B, QEA (E16Q, G18E, T85A) is shown to be linked to 487 T and V but not 487 A and L (and thereby not linked to the PSMVT/QCIGP haplotypes). In addition, T20S is linked to 487 V, whereas E24D+G27S is linked to 487 T (detailed data are shown in Fig. S3). The QE part of this N-terminal variation has been noted previously (9) but not these larger patterns of linkage. The T585I C-terminal variation is linked to 487V and is at the same amino acid position as the T of PSMVT. When the functional significance of these polymorphisms is explored experimentally, this understanding of the relationship of N- and C-terminal variation of EBNA1 will help focus analysis of EBNA1 polymorphism on combinations which are present in natural isolates of EBV. There is still no linkage between the type 1/type 2 classification and variation in EBNA1 (Fig. S3).

### SNPs reported to be linked to NPC.

**FIG 4:**
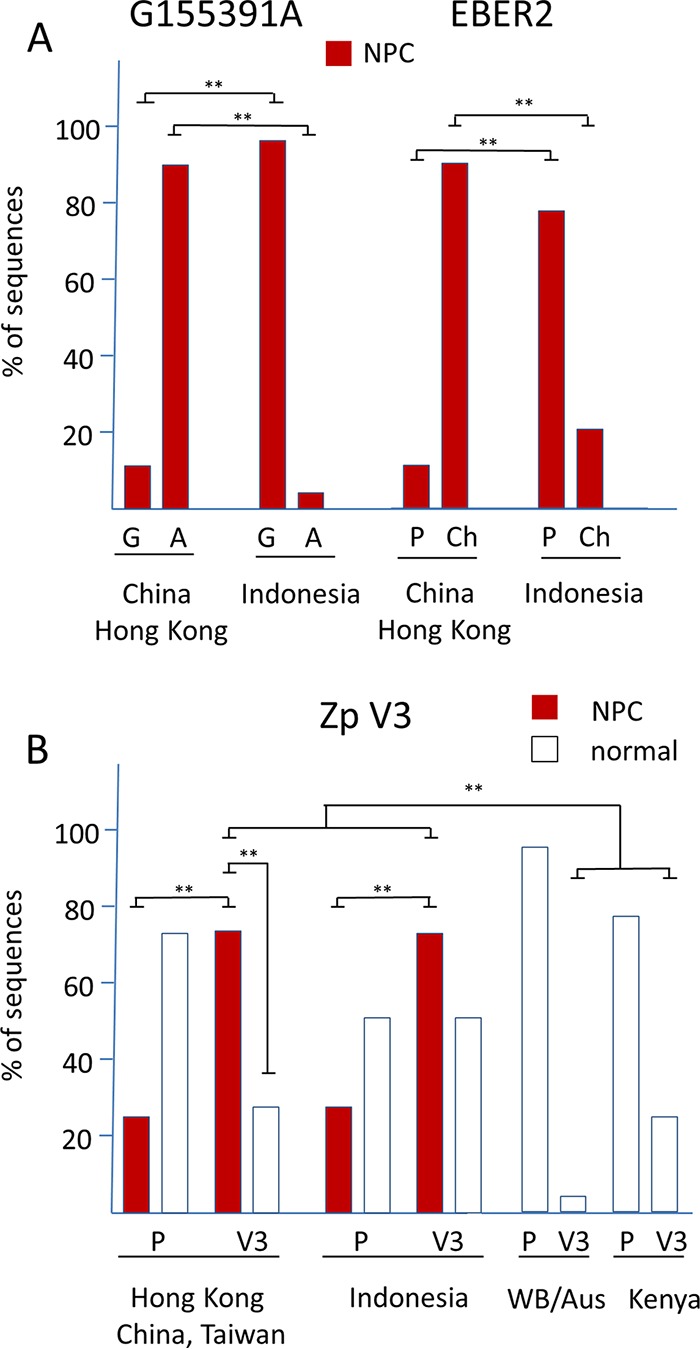
(A) Percentage of G55391A and EBER2 alleles in EBV sequences from NPC or normal samples from China/Hong Kong and Indonesia. Details of strains are shown in Fig. S4, sheets 1 and 2. Significant differences were determined with an ANOVA test. **, *P* < 0.005. (B) Percentage of Zp P and V3 alleles in EBV sequences from NPC or normal samples from Asia, Indonesia, White British (WB)/Australia, and Kenya. Details of strains are shown in Fig. S4, sheet 3. Significant differences are indicated. **, *P* < 0.005. The Indonesian samples (NPC and normal samples) were collected in and around Jakarta and have been described previously (35 and references therein).

The SNP G155391A in the RPMS1 open reading frame has been correlated with the high incidence of nasopharyngeal carcinoma (NPC) in southern China (10). Comparison of EBV sequences from North and South China identified this SNP; the consequent Asp-to-Asn amino acid change in the RPMS1 protein was found to reduce the stability of the protein (10), although uncertainty remains about whether the RPMS1 protein is expressed in EBV-infected cells (11). A further sequence study supported this distribution of the SNP selectively in southern China (12). In our data, 95% of NPC samples from China and Hong Kong had this G155391A SNP (Fig. 4A; detailed data are shown in Fig. S4, sheet 1). However, there is also an elevated frequency of NPC in Indonesia, but only 1 of our 20 Indonesian NPC cases had the G155391A SNP (5%) (Fig. 4A). Our results therefore support the high frequency of the G155391A SNP in EBV sequences from Hong Kong and southern China but question the more general relationship to NPC, since G155391A is only rarely present in NPC cases from Indonesia.

A more frequent SNP in the adjacent codon of the RPMS1 open reading frame occurs at C155389T and changes the RPMS1 amino acid Pro to Leu. Seventy of 237 isolates (29.5%) have this SNP (Fig. S4, sheet 1), which was relatively frequent in EBV from Indonesia and Papua New Guinea but could also be found in EBV from other geographic regions. There was no relationship of this SNP to disease in our data set.

Similarly, for EBER2 a polymorphism (named Ch; the prototype is designated P) has been reported that was linked to southern Chinese NPC strains (13). This region of EBER2 has the sequence GcTgtgcggtgctGccgTc in Ch isolates but is TcAgtgcggtgctAccgAc in the P strains (Fig. S4, sheet 2, lists strains classified by this polymorphism). Again, this polymorphism is frequent in southern Chinese NPC strains but has a much lower incidence in Indonesian NPC isolates (Fig. 4A), questioning its relationship to NPC.

Several previous studies have noted the V3 polymorphism in the Zp promoter (4, 14, 15, 16), and recent work has shown that this affects Zp function, the V3 form inducing BZLF1 expression, and the lytic cycle of EBV in lymphocytes more strongly (17). The V3 form of Zp was found to be enriched in the EBV present in African BL (17). The Zp V3 allele has also been shown to be overrepresented in NPC cases from Hong Kong containing type 1 EBV (18). This was confirmed in our data for Hong Kong, China, and Taiwan, which show a higher frequency of Zp V3 in the NPC cases than normal isolates in our study (Fig. 4B; detailed data are shown in Fig. S4, sheet 3). A high frequency of V3 was also present in the NPC cases from Indonesia compared to normal infections from other parts of the world (Fig. 4B). Although the percentage of V3 in NPC cases was also higher than that in normal infections from Indonesia (Fig. 4B), there were not sufficient Indonesian normal infections in our study to establish statistical significance of that aspect. The V3 version of Zp therefore correlates more generally with NPC incidence in our data set than the RMPS1 or EBER SNPs discussed above, perhaps consistent with the EBV lytic cycle gene expression observed in NPC biopsy specimens and high antibody titers to EBV lytic cycle antigens observed in NPC patients.

The LMP2A gene of EBV is also expressed in NPC, and a novel polymorphic region in LMP2A was identified by examining phylogenetic trees of the N-terminal LMP2A protein sequence encoded in its first exon (the part involved in signal transduction) from 203 EBV strains in the MSA. The reference NC_007605 LMP2A protein sequence of amino acids 63 to 82 (PYwgngdrhsdyqplgTqdQ) varies at the positions shown in uppercase from the reference PYTQ (84 strains) to PYTP (32 strains), PDNP (48 strains), and LDNP (39 strains). All of the NPC isolates are in the PYTQ and PYTP groups. The Y of PYTP (Y64 of LMP2A) is not thought to be phosphorylated in LCLs, but the proline amino acid changes are likely to have structural effects and are close in the protein sequence to the functionally important PY1 motif (19), which is amino acids 56 to 60. It would be interesting to examine functional effects of these sequence differences, with the most variant form (LDNP) being found mainly in Hodgkin's lymphoma and PTLD samples of European origin in our data set.

### Codon usage differs between latent and lytic cycle EBV genes.

**FIG 5:**
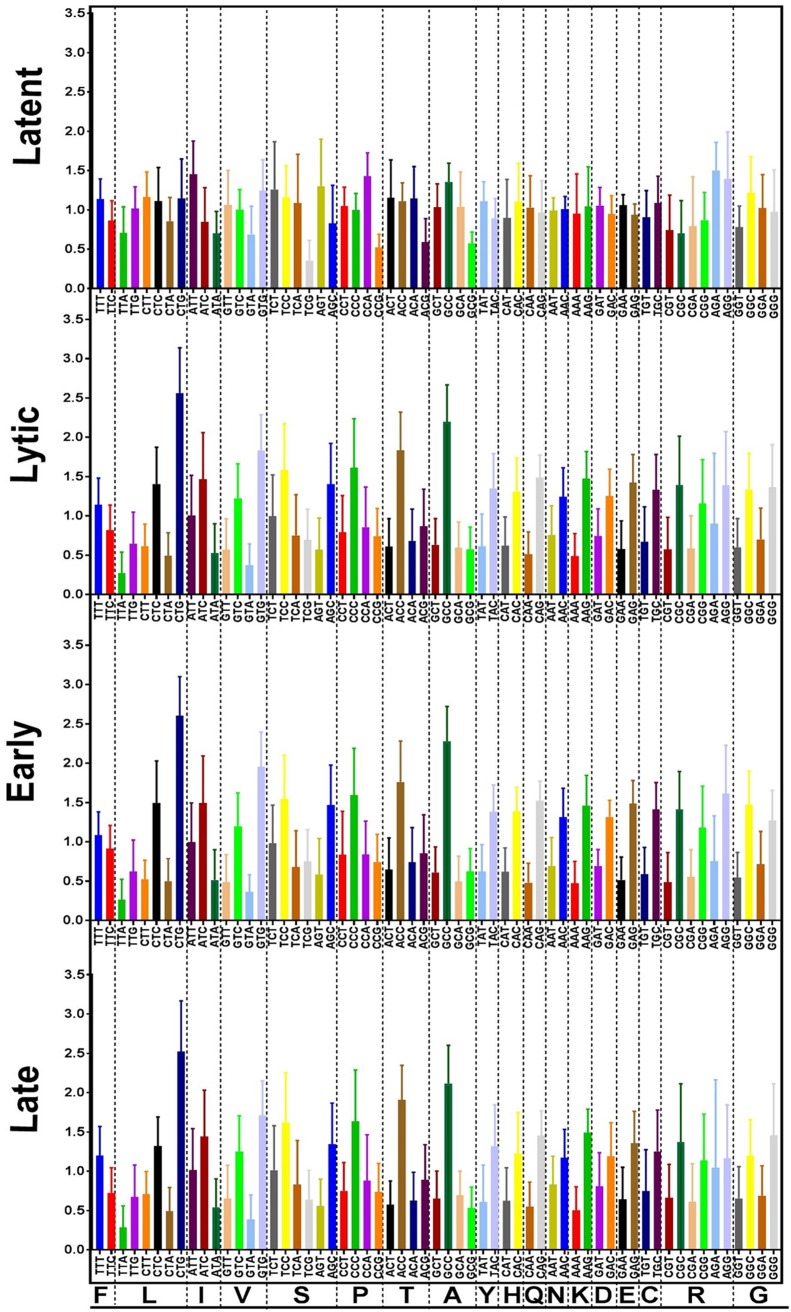
Relative synonymous codon usage for combined open reading frames of NC_007605, AG876, and Akata EBV. (Upper) Latent and lytic cycle genes. (Lower) Lytic genes separated into early and late groups. Detailed values and results for each strain analyzed separately are shown in Fig. S5.

We have now extended our previous analysis of positively selected amino acids (5) into a more general comparison of codon usage of latent and lytic cycle EBV genes. In several viruses, including, for example, human papillomaviruses (HPVs), variations in codon usage have been linked to gene expression. Differences in codon usage in late HPV genes compared to the early genes have been related to variations in tRNA pools available for translation in the more differentiated layers of epithelium where late gene expression occurs (20). We used the reference EBV NC_007605 (a chimera of U.S. and African strains), Akata (Japanese), and AG876 (type 2) sequences for our analysis, and Fig. 5 shows the combined results. The glycine/alanine repeat region of EBNA1 was excluded to avoid it skewing the results for those codons. There are clear differences in codon preference between the latent cycle genes of EBV and the lytic cycle genes. Codon usage is more evenly spread in the latent cycle genes than the lytic cycle genes, which show peaks of higher use of specific codons (Fig. 5).

In the representation of codon usage in Fig. 5, the values would all be 1 if all codons were used equally. The statistical significance of the difference between latent and lytic cycle genes shown in Fig. 5 was quantified by comparing the differences for each codon to a value of 1 (data are shown in Fig. S5, sheet 3, strains combined). The variation in codon usage was shown to be significantly greater in the lytic cycle genes than in the latent cycle genes (*P* value of <0.0001 in an unpaired *t* test). Separation of the lytic cycle genes into early and late groups showed no difference between these two groups (Fig. 5), and each strain gave similar results when analyzed separately (Fig. S5).

Since latent persistence of EBV occurs in B lymphocytes but lytic replication may be adapted to the suprabasal layers of stratified epithelium (21), it would be interesting to determine whether the different codon usage patterns are related to differences in tRNA populations in these cell types. Because the three strains analyzed (NC_007605, Akata, and AG876) all derived from lymphoid infections, a similar analysis was done for the GD2 EBV strain, directly sequenced from the epithelial cell infection in an NPC case. The results (Fig. S5, sheet GD2) were very similar to those shown in Fig. 2 for the three lymphoid isolates.

### Heterogeneity of EBV isolates.

Our previous PCR analysis of saliva for type 1 and type 2 EBV (4) showed many examples of both types of EBV in single samples, confirming that people can carry multiple strains of the virus. Sequence assembly software is designed to produce a single unique assembled sequence where possible, and this can make it difficult to recognize mixtures of sequences; mixtures may even result in a failure to assemble *de novo* a complete sequence, even though there are many sequence reads.

**FIG 6:**
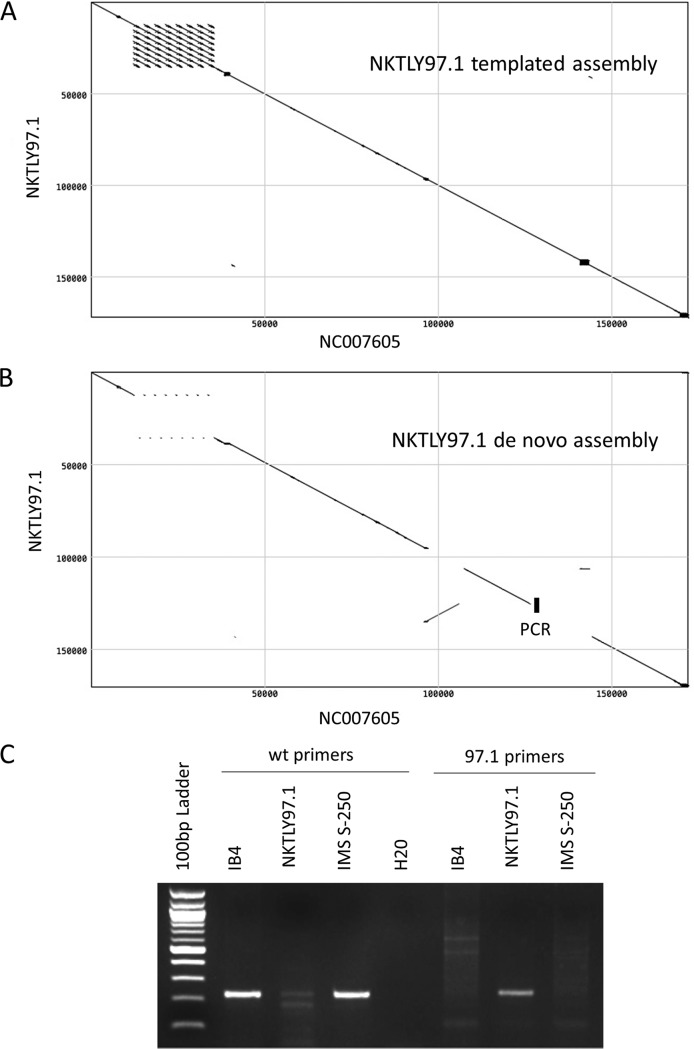
(A and B) Dot matrix plots of similarity between NKTLY97.1 and NC_007605 EBV sequences (window size, 30; minimum score, 85%; hash value, 8; MacVector). Templated BWA assembly of NKTLY97.1 (A) and *de novo* assembly of NKTLY97.1 (B) were used. (C) Agarose gel electrophoresis of products from PCR across the novel sequence boundary marked in panel B is shown with the 97.1 primers. PCR with WT primers (see Materials and Methods) is shown on the left.

An example of a tendency of templated assembly to bias the result toward the template sequence comes from our sequencing of the NKTLY97.1 NKT lymphoma cell line (Table S1, omitted from the MSA of 241 sequences). Here, the BWA templated assembly sequence was similar to the reference type 1 EBV (NC_007605), showing a simple diagonal line on the dot matrix plot (Fig. 6A). The clustered signals from the major internal repeat array and other repeats are visible, as expected (Fig. 6A). In contrast, the *de novo* assembly dot matrix plot showed a section of the NKTLY97.1 genome around position 100,000 to be inverted, as well as some other differences (Fig. 6B). To ensure the inversion was not a sequence assembly error, PCR primers were designed to span the predicted sequence at the right-hand end of the inversion. These gave a clear product of the expected size with the NKTLY97.1 DNA but not with control EBV DNA from the IB4 cell line or from a saliva sample, IMS 250 (Fig. 6C), confirming the sequence inversion. The PCR product from the rearranged genome was sequenced, confirming its identity and giving the breakpoint sequence as GGTTGGCGTAGCAGGAGGCA/ACTGCTACGGGGGCG/CTCGTCTTCGCTCTTGGCC. The central ACTGCTACGGGGGCG section of sequence inserted at the breakpoint did not align with EBV genome on either side of the breakpoint. PCR of the equivalent region with primers for the standard EBV genome gave a product with IB4 and the saliva sample but also a much weaker signal from the NKTLY97.1 DNA. We conclude (Fig. 6C) that NKTLY97.1 contains mostly the rearranged form of the EBV genome (which dominates in the *de novo* assembly) but sufficient standard EBV genome to assemble into the standard form with the templated assembly.

Eight of the EBV samples we sequenced and included in the MSA came from saliva of chronic active EBV infection (CAEBV) patients from the United States (22). We investigated the possibility that these samples also include some rearranged EBV genomes similar to the het EBV that was found previously to be the basis of the chronic lytic cycle activation in the P3HR1 cell line (23, 24). The het EBV is a rearranged defective genome in which the latency Wp promoter is juxtaposed (WZhet) to the BZLF1 gene, which induces the lytic cycle. The *de novo* assemblies of these CAEBV patient sequences did not show rearranged EBV genomes, but typically not all of the contigs are used in the final assembly. We therefore examined all other contigs that were obtained for these samples for sequences from the start of the BZLF1 open reading to 500 nt upstream, which would be characteristic of the het rearrangement (see Materials and Methods for details). All of the contigs omitted from the final sequence assembly that contained any part of this Zp sequence aligned to the standard EBV genome. The contigs were also examined for Wp sequence between the TATA box to 300 nt downstream, but again there were no such contigs that were not colinear with the standard genome. Finally, for these samples we also examined the sequence reads that had failed to map to their assembled genome. We searched these reads for ones that either aligned to the WZhet junction or included any other junction between the start of the BZLF1 open reading frame to 500 nt upstream and the Wp from the TATA box to 300 nt downstream. Within these sequence positions we did not find any evidence for a WZhet type of EBV genome rearrangement in the saliva of these CAEBV patients.

## DISCUSSION

The purpose of this study was to create an expanded set of EBV genome sequences sufficient to identify major patterns of variation that can, in future work, be investigated functionally and tested for correlation with disease. Although many examples of EBV from cell lines have been sequenced, most of the new sequences we report here come from primary material, including saliva samples from healthy carriers, cancer samples, and other diseases. EBV genomes from many different parts of the world are included in this new data set, giving some insight into the degree of variation that may be expected worldwide. This is the most comprehensive analysis of worldwide EBV sequence variation reported so far.

Although the unique regions comprising most of the EBV genes have largely been fully sequenced, the many repeat regions within the EBV genome remain a problem for sequence assembly. The relatively short sequence reads obtained with Illumina sequencing generally make it difficult to allocate reads uniquely to any individual repeat, although some progress has been made with the major internal repeat (25). Alternative sequencing methods with much longer reads may be able to circumvent that limitation, but they are not compatible with the enrichment protocols that were necessary to allow the sequencing of EBV from primary infected cells described in this paper. Those alternative methods are, however, useful for complete sequencing of EBV BAC clones (26).

Another complication of Illumina sequencing EBV from primary material is the presence of mixtures of strains. This was clearly illustrated in our analysis of the NKTLY97.1 lymphoma and was also implied by the frequency of coinfection with type 1 and type 2 EBV in saliva samples collected from healthy students in our previous study (4). Deconvoluting the sequence reads into multiple strains remains a complicated matter, and mixed sequences may explain why some of the samples we analyzed (see Table S1 in the supplemental material, MG298917-28) did not produce sufficiently long contigs by *de novo* assembly to allow the EBV genome to be properly assembled, even though there were many sequence reads mapping to EBV. Those were primary isolates from saliva or tonsils of healthy donors or saliva of chronic active EBV infection patients.

Sequence heterogeneity of EBV during acute infectious mononucleosis has been analyzed recently, showing that the heterogeneity reduced as the disease resolved to a chronic infection (27). Eleven of the strains we sequenced were from Japanese infectious mononucleosis patients (Table S1, 164 to 179 of the 241 aligned strains). Those assembled sequences have some significant stretches of Ns in them, which can arise from heterogeneity, and the European Nucleotide Archive (ENA) references for those sequence reads are given in Table S1 to allow further investigation of that point.

In contrast, the EBV sequences from saliva of the majority of cases of chronic active EBV infection patients assembled well, giving only standard EBV genome structures and no evidence for the suspected WZhet type of rearranged genome. The sheared DNA fragments that were enriched by the Sureselect method and sequenced were at least 500 bp in length, and the RNA oligonucleotides on the Sureselect beads were 120 nt long. Therefore, wherever the breakpoint might occur in the DNA fragment there would be at least 120 nt to anneal with a Sureselect oligonucleotide from either the Wp or Zp side. Thus, we do not think that potential WZhet type rearranged DNA would have been omitted from the enriched DNA that was sequenced. The results suggest that these CAEBV cases are due to immunological or host genetic changes or other failures in control of EBV rather than coinfection with a mutant EBV strain, similar to WZhet EBV (22). It should be noted that in this study we only investigated the EBV secreted in saliva from CAEBV cases and not the EBV present in cells in the blood or other organs.

Principal-component analysis of SNP distribution clearly identifies the major type 1/type 2 distinction and clusters sequences from geographic regions. Our attempts to relate variation in EBV genome regions to be characteristic of specific clusters of strains were successful with the comparison of type 1 and type 2 but less clear with other forms of geographic variation. Consistent with our earlier analysis of 84 EBV genomes, the type 1/type 2 difference is mainly accounted for by variation in EBNA2 and the EBNA3 locus, but the type 2 character of the EBNA3 region extends beyond the EBNA3 genes into the N terminus of gp350 on one side and through gp42 to Zp on the other side. In view of the recent discovery that type 2 EBV also may be able to infect T cells (28, 29), it will be important to test the effects of the variation in gp350 and gp42 in a controlled system comparing type 1 and type 2 BAC EBV clones in which gp350 and gp42 can be manipulated. The clear genetic linkage between EBNA2 and EBNA3, even though the genes are 60 kb apart on the genetic map, also implies a currently unknown mechanism that may relate to a functional compatibility between the proteins. Since the positively selected amino acids in EBNA3 were not restricted to T cell epitopes (5), it seems that the selection that links EBNA2 and EBNA3 is not due to immune surveillance but due to functional compatibility. This could be investigated using BAC EBV clones engineered to contain the different combinations of type 1 and type 2 EBNA2 and EBNA3.

The improved understanding of EBNA1 variation that has come from this analysis will facilitate testing for effects on EBNA1 function. A recent study (30) addressed this for two PSMVT C-terminal regions and found only modest effects but did not address the major QCIGP variant or the linked variation in the N terminus of the protein. Our results define the main forms of the EBNA1 protein that occur in normal and cancer cells and will allow future studies to focus on the most prevalent EBNA1 sequences.

The differences in codon usage between latent and lytic cycle EBV genes demonstrated here indicate evolution of the gene sequences to match tRNA populations in the cells where these phases of EBV occur in the human body. Latent infection in lymphocytes and lytic infection in epithelial cells would be one possibility for EBV. This type of codon usage preference has not been reported yet for other herpesviruses, partly because of the very few proteins expressed in the latent infections of most herpesviruses.

The extension of the PCA and SNP procedures to include insertions and deletions made the analysis more susceptible to alignment weaknesses within the MSA but increases our confidence that there are no other unrecognized regions of the EBV genome yet to be discovered. We succeeded in identifying a previously unrecognized insertion in some strains within the BART microRNA (miRNA) region. This insertion might affect the processing of miRNAs, as even quite distant mutation of the BART miRNA region has been found to affect miRNA levels (4). A more detailed investigation of the SNPs that correspond to individual principle components of variation and the geographic distribution will be published elsewhere. Although the PCA clearly separated type 1 sequences from type 2, at least the first 3 components contributed to that separation (Fig. 1A). There was not, for example, a simple correspondence between PCA1 and EBNA3 variation and PCA2 and EBNA2 variation. It seems that the components reflect complex overlapping sets of SNPs distributed along the genome. This suggests some more fundamental selective pressures that operate on the evolution of this viral genome, most likely combinations of multiple immunological and functional selective mechanisms.

## MATERIALS AND METHODS

### EBV DNA sequencing and sequence assembly.

Sample preparation, enrichment of EBV DNA, and sequencing were as described previously (5), except sequencing was on the Illumina HiSeq 2500, with paired-end reads of 250 nt generating fastq files. Reads were quality controlled using QUASR (5).

For initial BWA mapping (version 0.7.12) (31) to produce templated sequence assemblies, all reads were mapped against NC_007605. Type 2 strains were also mapped against AG876 (NC_009334) to generate consensus sequences. An alignment of these templated consensus sequences was used to produce the phylogenetic tree shown in Fig. S1 in the supplemental material.

For *de novo* assembly, SPAdes assembler (version 3.5.0) (32) was used to generate files of contigs for each sample. The SPAdes contigs were then run through scripts to improve the assembly by remapping the ends of the contigs. The resulting contigs were filtered to use only those over 500 bp, and for each sample the contigs from SPAdes were mapped using Geneious (www.geneious.com) against the reference sequences NC_007605 and NC_009334 for type 2 strains. Contigs were also mapped to the BWA consensus for that sample. These multiple-sequence alignments of contigs were then used to create a consensus which was the *de novo*-assembled genome sequence.

Samples ebv6 to ebv15 were processed using CLC Genomics Workbench 7, including the CLC Microbial Genome Finishing Module (Qiagen). EBV-specific reads, obtained by mapping against a database of previously available EBV genomes, were *de novo* assembled into contigs and merged. Reads were mapped back to the contigs and a consensus sequence extracted with a minimum depth threshold of 20.

Samples ebv16 to ebv31, P(1-4)-T1, and the Japanese infectious mononucleosis samples (LS992259 to LS992269) were trimmed using TrimGalore (v0.3.7; Babraham Bioinformatics) with a quality threshold of 20 and then aligned with BLASTN (U.S. National Library of Medicine) against database type 1 and 2 EBV genomes. The EBV-specific reads were *de novo* assembled using SPAdes v3.5.0 (32) into contigs. Those with a length of >200 bp were used to generate a scaffold with an in-house R-script. Reads were remapped against the scaffold with BBMap (Joint Genome Institute) and further processed with SAMtools, v.0.1.19 (Genome Research Ltd.), and Picard (Broad Institute). The consensus was extracted using QUASR (Bioconductor) with a minimal depth threshold of 20 and a minimal base quality of 20.

### Multiple EBV sequence alignment and PCA.

The 138 new *de novo*-assembled EBV sequences and published EBV sequences (241 in total) were aligned using MAFFT (33), with some manual editing to reduce gapping in repeat sequence alignments. PCA was performed with custom Delphi/Pascal software using a single-value decomposition library from SDL Component Suite (http://www.lohninger.com). Details of the MSA and further analysis of geographic variation will be published separately.

### Codon usage analysis.

Genome sequences corresponding to annotated open reading frames in the NC_007605 reference EBV sequence, AG876 (NC_009334), and Akata (KC207813) EBV were assembled manually as fasta files. The Gly-Ala repeat of EBNA1 was omitted from the latent cycle protein sequences used for this analysis to avoid bias. Relative synonymous codon usage was then determined using the codon usage utility in the SMS programs (34) at http://www.bioinformatics.org/sms2/codon_usage.html. Results were combined in Excel (Fig. S8) and plotted using GraphPad Prism (Fig. 5).

### PCR of rearranged sequence in NKTLY97.1 DNA.

Primers for the standard EBV genome were GAAAATTCTTGAGCCGGC and ACATAATGGATGGGCAGG, and primers for the rearranged genome were TACTGCGAAGGGAAGATG and ACATAATGGATGGGCAGG. PCR was 30 cycles with an annealing temperature of 49°C.

### CAEBV samples.

Samples from patients with CAEBV were obtained from patients at the National Institutes of Health who signed consent for a protocol that was approved by the Internal Research Board of the National Institute of Allergy and Infectious Diseases. For analysis of EBV genomes in CAEBV cases, contigs were searched on both strands for CAAAGATAGC, CAAGGTGCAATGTTT, ATCTCCCCTTTAAAG, GGTTTGGGACGTGCTAAATTT, TAAAATAAGCTGGTGTCAAAAAT, TCAGGGGGGAGTCCAGATTC, and TATGGCTGCTTCCTCCTTCTG. These were Zp sequences spread between the start of the BZLF1 open reading to 500 nt upstream that were conserved in the MSA of this part of the genome. Contigs were also searched on both strands for CCCCCTCCCTAGAA, TAAACGCGCTGGACTGAGAA, and GGTGAAGTCACAAACAAGCCC from Wp, these sequences lying in the region between the TATA box to 300 nt downstream.

The sequence reads from the CAEBV cases were also remapped onto their corresponding consensus sequence using the BWA mem aligner, and reads that did not map were then searched using the sequences shown above, which would identify reads with translocations between Wp and Zp. Additionally, reads that did not map were remapped against the WZhet sequence to check for alignments. Finally, the CAEBV sequence reads were also mapped using a lower-stringency aligner (BWA aln) against the NC_007605 reference sequence, and the reads that did not align were mapped on to the WZhet sequences (M20820.1 and M33474.1).

### Accession number(s).

Sequences determined in the course of this work have been deposited in GenBank, and their accession numbers are listed in Table S1.

## Supplementary Material

Supplemental file 1

Supplemental file 2

Supplemental file 3

Supplemental file 4

Supplemental file 5

Supplemental file 6
