# Supplementary figures and images for: Sequence Variation of Epstein-Barr Virus: Viral Types, Geography, Codon Usage, and Diseases
Source: J Virol. 2018 Oct 29;92(22):e01132-18. doi: 10.1128/JVI.01132-18 (PMC6206488; doi:10.1128/JVI.01132-18)

Fig S1  
Whole genome alignment of  
233 EBV sequences

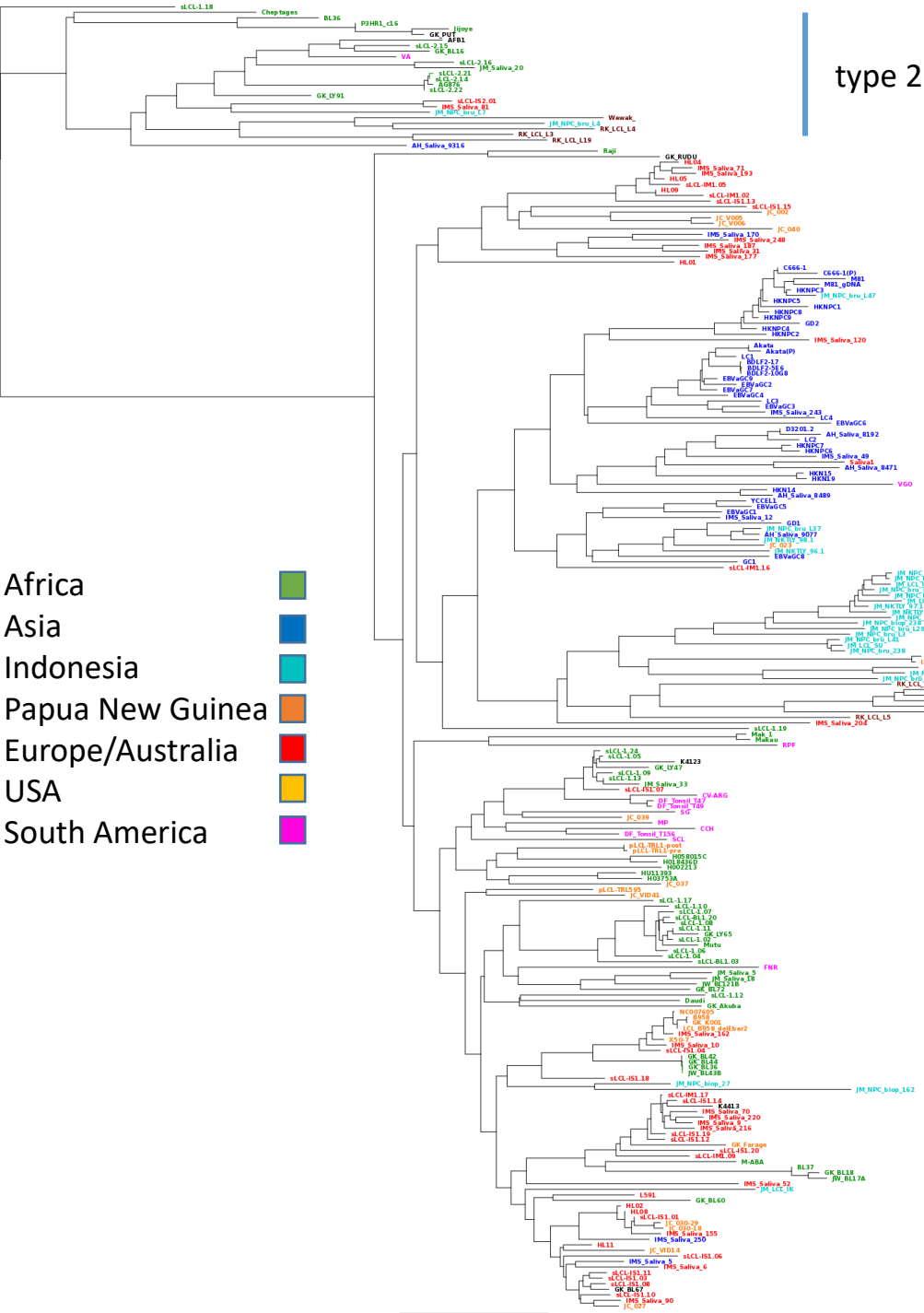

Supplement: Supplemental file 1 [file zjv021183971s1.pdf]
